# Editorial: Emotional Dysregulation in Children and Adolescents

**DOI:** 10.3389/fpsyt.2022.883753

**Published:** 2022-04-07

**Authors:** Eva Moehler, Romuald Brunner, Carla Sharp

**Affiliations:** ^1^Department of Child and Adolescent Psychiatry, Saarland University Hospital, Homburg, Germany; ^2^Department of Child and Adolescent Psychiatry, University of Regensburg, Regensburg, Germany; ^3^Department of Psychology, University of Houston, Houston, TX, United States

**Keywords:** emotion, self-regulation, disruptive mood dysregulation, emotion regulation, Emotion Dysregulation

Emotional Dysregulation (ED) is defined as the inability to regulate and organize emotions to produce an appropriate emotional response and subsequent return to baseline. With regard to the intensity of reactions it partially overlaps with the concept of irritability (1), which is however regarded to be a more dispositional trait. ED represents a major health risk (2) and is associated with diverse forms of childhood psychiatric disorders and symptoms like attention deficit hyperactivity disorder, oppositional defiant- and conduct disorders (ODD and CD), personality disorders, self-injurious behavior and suicidality. In clinical settings, dysregulation problems are especially prominent (3), occurring in 26.0–30.5% of children admitted to child and adolescent psychiatric clinics or mental health facilities. A recent study could demonstrate that especially disturbed emotion regulation contributes to self-injurious behavior in a large group of adolescents presenting to a child and adolescent psychiatric emergency service (Kandsperger et al.). The occurrence of typical phenomena associated with ED, like severe tantrums, low frustration tolerance, aggression, negative mood and suicidality is even higher than the full syndrome of ED, with estimates of about 45% in child psychiatric patients between 6 and 18 years (4). An additional impact of Lockdown-related stressors on children and adolescents can also be discussed [e.g., (5)]. Thirty percentage of adults with emotional instability report having injured themselves at primary school age (6).

Several authors also mention emotionally dysregulated behavior as one of the leading symptoms of BPD and ADHD/DMDD (7). On the opposite note, emotion regulation capacity prevents the onset of multiple psychiatric and physical disorders and promotes successful social and professional development as described above (8). In connection with Emotion Dysregulation, large and rigorous studies (6) also emphasize that this behavior results in high costs for health and other services services.

In addition to biological vulnerability, epidemiological research suggests that the onset of most psychiatric disorders across the life course in nearly half of cases is attributable to adverse childhood experiences and stress related disorders (9, 10). Negative impact of adverse childhood experiences on general health seems to attributable partly to maladaptive strategies for emotion regulation, such as smoking, alcohol, overeating (11). Sleep problems related to the traumatic impact of adverse childhood experiences can be directly related (10, 12) and be an important target for treatment. Furthermore, early life trauma impairs neurobiological structures and functions related to emotion regulation, such as the orbitofrontal gyrus and frontolimbic connections (13). A challenge for clinical practice is the assumption that patients with ED remain notoriously difficult to treat (14).

Better equipment with psychotherapeutic tools for Emotion Regulation and characterization of the ecological contingencies, and an understanding of the developmental pathways through which early experience shapes later behavior, can help clinicians to tailor intervention efforts more precisely, to prevent future dysfunction (15).

Therefore, studies focusing on pathogenetic aspects of ED by addressing neurobiological underpinnings and childhood adversity are collected in this issue. Furthermore, interventions and therapies that give an overview on established therapeutic tools such as DBT and the younger “derivatives” and describe novel interventions developed from the recent ED-Framework, are included in this topic. Together with review articles on state of the art advancements in ED, research in this issue explores the adverse childhood experiences framework or describing empirical research on neurobiological associations.

With original articles and reviews on diagnosis and classification of ED our aim was to achieve with this issue a large transdiagnostic long-term benefit for research as well as clinical aspects since understanding and improving human emotion regulation capacity prevents the onset of multiple psychiatric and physical disorders and promotes successful social and professional development as will be shown in this issue.

## Author Contributions

All authors listed have made a substantial, direct, and intellectual contribution to the work and approved it for publication.

## Conflict of Interest

The authors declare that the research was conducted in the absence of any commercial or financial relationships that could be construed as a potential conflict of interest.

## Publisher's Note

All claims expressed in this article are solely those of the authors and do not necessarily represent those of their affiliated organizations, or those of the publisher, the editors and the reviewers. Any product that may be evaluated in this article, or claim that may be made by its manufacturer, is not guaranteed or endorsed by the publisher.
